# A Modified Tri-Exponential Model for Multi-*b*-value Diffusion-Weighted Imaging: A Method to Detect the Strictly Diffusion-Limited Compartment in Brain

**DOI:** 10.3389/fnins.2018.00102

**Published:** 2018-02-26

**Authors:** Qiang Zeng, Feina Shi, Jianmin Zhang, Chenhan Ling, Fei Dong, Biao Jiang

**Affiliations:** ^1^Department of Neurosurgery, Second Affiliated Hospital of Zhejiang University School of Medicine, Hangzhou, China; ^2^Department of Neurology, Second Affiliated Hospital of Zhejiang University School of Medicine, Hangzhou, China; ^3^Department of Radiology, Second Affiliated Hospital of Zhejiang University School of Medicine, Hangzhou, China

**Keywords:** diffusion magnetic resonance imaging, brain, white matter, computer-assisted image processing, theoretical models

## Abstract

**Purpose:** To present a new modified tri-exponential model for diffusion-weighted imaging (DWI) to detect the strictly diffusion-limited compartment, and to compare it with the conventional bi- and tri-exponential models.

**Methods:** Multi-*b*-value diffusion-weighted imaging (DWI) with 17 *b*-values up to 8,000 s/mm^2^ were performed on six volunteers. The corrected Akaike information criterions (AICc) and squared predicted errors (SPE) were calculated to compare these three models.

**Results:** The mean *f*_0_ values were ranging 11.9–18.7% in white matter ROIs and 1.2–2.7% in gray matter ROIs. In all white matter ROIs: the AICcs of the modified tri-exponential model were the lowest (*p* < 0.05 for five ROIs), indicating the new model has the best fit among these models; the SPEs of the bi-exponential model were the highest (*p* < 0.05), suggesting the bi-exponential model is unable to predict the signal intensity at ultra-high *b*-value. The mean *ADC*_*very*−*slow*_ values were extremely low in white matter (1–7 × 10^−6^ mm^2^/s), but not in gray matter (251–445 × 10^−6^ mm^2^/s), indicating that the conventional tri-exponential model fails to represent a special compartment.

**Conclusions:** The strictly diffusion-limited compartment may be an important component in white matter. The new model fits better than the other two models, and may provide additional information.

## Introduction

Diffusion-weighted imaging (DWI) is the only noninvasive method for detecting the diffusion motion of water molecules in tissues. Currently, in clinical practice, apparent diffusion coefficient (ADC) maps are typically calculated with the mono-exponential model. However, it has been well established that the attenuation of the DWI signal does not follow the mono-exponential model in many tissues (Clark and Le Bihan, 2000; Koh et al., 2011). It is believed that more than one proton pool exists inside each voxel (Bennett et al., 2003, 2006). Therefore, many models have been developed in order to accurately reflect the diffusion motion of water molecules *in vivo* (Le Bihan et al., 1988; Bennett et al., 2003; Jensen et al., 2005), such as the bi-exponential model.

However, the bi-exponential model is being challenged for the heterogeneity of its results across studies and its lack of reproducibility (Müller et al., 1998; Grant et al., 2001; Schwarcz et al., 2004; Koh et al., 2011; Steier et al., 2012; Hu et al., 2014; Lin et al., 2015). This model is considered oversimplified (Bisdas et al., 2013), and some researchers even speculate that there are continuous distributions of diffusion coefficients inside each voxel (Bennett et al., 2003, 2006). Recent studies have demonstrated that the three-pool model can perform a better fitting and may provide more detailed information than the bi-exponential model in many tissues (Hayashi et al., 2013, 2014; Cercueil et al., 2015; Ohno et al., 2016; Ueda et al., 2016; van Baalen et al., 2017). However, it is well known that a highly parameterized model can always fit a given data set better than a model with fewer parameters. Whether or not a model can provide more information should be well verified to avoid over-fitting.

Recently, ultra-high *b*-value DWI has been studied more frequently in recent years because of the popularization of 3.0-Tesla MR systems (Ling et al., 2015; Hu et al., 2017). Several studies using DWI with ultra-high *b*-value have found that the signal curves of DWI decrease very slowly and tend to be stability at ultra-high *b*-values in some tissues (Grant et al., 2001; Ling et al., 2015). This phenomenon can be hardly explained by the existing models. Many studies have suggested the existence of the strictly diffusion-limited compartment with extremely low ADC in tissues and even cells (Niendorf et al., 1996; Grant et al., 2001; Sen and Basser, 2005; Baxter and Frank, 2013; Ling et al., 2015). The ADC of water molecules, which are strictly limited in microstructures with extremely small space (such as intracellular organelles), might be extremely low. Because of the very low signal attenuation of this compartment at normal *b*-values, the ADC of this compartment can be set as zero mathematically. Accordingly, by setting one ADC value of the conventional tri-exponential model as zero, we developed a new modified tri-exponential model.

The purpose of this study was to present the new modified tri-exponential model to detect the strictly diffusion-limited compartment, and to compare it with the conventional bi- and tri-exponential models. Previously, stationary water molecules have been suspected to be exist in white matter (Alexander et al., 2010), and three compartment models with a “dot” compartment (zero radius sphere) have been found to produce a better fitting for diffusion MRI in white matter (Ferizi et al., 2014). Accordingly, we hypothesized that the modified tri-exponential model with a “zero-ADC” compartment might also produce a better fitting for DWI in white matter. Hence, we performed this preliminary study in brain. To indicate the existence of the strictly diffusion-limited compartment, a multi-*b*-value DWI sequence with *b*-values range from 0 to 8,000 s/mm^2^ was used in this study.

## Materials and methods

### Participants

This study was approved by the institutional review board at the Second Affiliated Hospital of Zhejiang University. Six young healthy volunteers (four males and two females, age range 24–27 years) were enrolled in this study, without any previous history of central nervous system diseases. Written informed consents were obtained from all participants. This study was conducted according to the principles expressed in the Declaration of Helsinki.

### DWI parameters

The volunteers were imaged on a 3.0-Tesla MR system (Discovery MR750, GE Healthcare Systems, Milwaukee, WI) with a gradient strength of 50 mT/m using an eight-channel high-resolution receiver head coil. A single-shot echo planer imaging sequence was used for the imaging with the following parameters: number of sections, 24; section thickness, 4 mm; field of view, 240 × 240 mm; matrix, 256 × 256; in-plane resolution, 0.94 × 0.94 mm; repetition time/echo time, 3,000/107.7 ms; phase FOV, 1.00; flip angle, 90; and pixel bandwidth, 1953.1 Hz/pixel. The sequence was performed with 17 *b*-values (0, 10, 20, 30, 50, 70, 100, 150, 200, 300, 500, 700, 1,000, 2,000, 3,000, 5,000, and 8,000 s/mm^2^) in three orthogonal directions, and the signals were averaged over three directions by the imaging system automatically. The numbers of scan averages (NSAs) for *b* = 0 to 8,000 s/mm2 were 1, 3, 3, 3, 3, 3, 3, 3, 3, 3, 3, 5, 5, 5, 5, 9, and 12, respectively. Magnitude reconstruction was applied by the imaging system automatically. The total scan time was 21 min 30 s.

### Models

**Figure 1:**
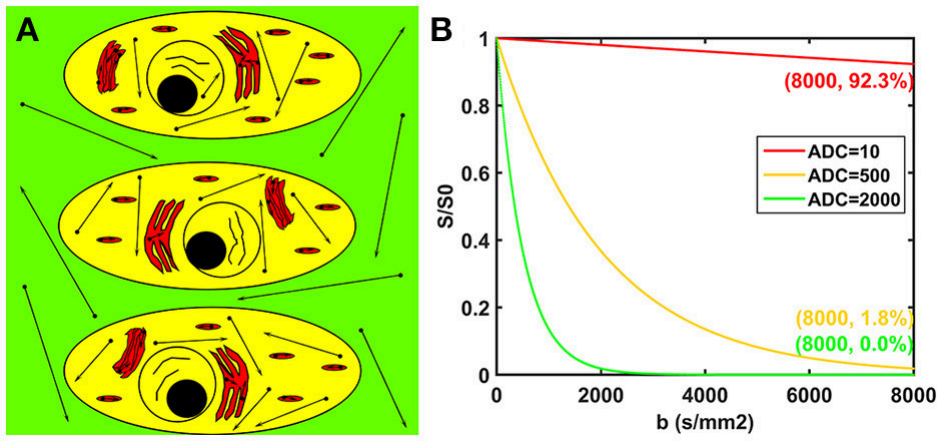
Potential biological interpretations for the three compartments of the modified tri-exponential model **(A)**. The red regions represent the strictly diffusion-limited compartment, the yellow regions represent the slow diffusion compartment, and the green regions represent the fast diffusion compartment. Signal attenuation curves of three ADC values **(B)**. The unit of the ADC values was mm^2^/s.

The potential biological interpretations for the three compartments of the modified tri-exponential model are shown in Figure 1A. The strictly diffusion-limited compartment is suspected to represent water molecules strictly limited in microstructures with extremely small space, such as intracellular organelles. The signal attenuation curves for three different ADC values are shown in Figure 1B. For normal ADC values, the remaining signal ratio will be very low at *b* = 8,000 s/mm^2^ (1.8% for ADC = 500 mm^2^/s and 0.0% for ADC = 2,000 mm^2^/s). However, the signal ratio will still remain high at *b* = 8,000 s/mm^2^ for an extremely low ADC value (92.3% for ADC = 10 mm^2^/s).

The signal attenuation of the bi-exponential model, the conventional tri-exponential model and the modified tri-exponential model as a function of *b* is expressed by Equations (1–3), respectively.

(1) $$\frac{S}{S_{0}}=f_{slow}*e^{-ADC_{slow}*b}+f_{fast}*e^{-ADC_{fast}*b},\;f_{slow}+f_{fast}=1$$

(2) $$\begin{array}{l} \frac{S}{S_{0}}=f_{very-slow}*e^{-ADC_{very-slow}*b}+f_{slow}*e^{-ADC_{slow}*b}\\ \;+f_{fast}*e^{-ADC_{fast}*b},\;f_{very-slow}+f_{slow}+f_{fast}=1 \end{array}$$

(3) $$\begin{array}{l} \frac{S}{S_{0}}=f_{0}+f_{slow}*e^{-ADC_{slow}*b}+f_{fast}*e^{-ADC_{fast}*b},\;f_{0}\\ \;+\;f_{slow}+f_{fast}=1 \end{array}$$

In these equations, *S* represents the signal intensity at corresponding *b*, and *S*_0_ represents the signal intensity at *b* = 0 s/mm^2^. The *f*_*very*−*slow*_, *f*_*slow*_, and *f*_*fast*_, respectively represent the fractions of corresponding diffusion compartments, with corresponding ADC marked as *ADC*_*very*−*slow*_, *ADC*_*slow*_, and *ADC*_*fast*_. The *f*_0_ represents the fraction of the strictly diffusion-limited compartment.

### Model ranking

For model selection, the small-sample corrected Akaike information criterion (AICc) has been widely used in previous studies (Bourne et al., 2014; Jambor et al., 2015). The akaike information criterion (AIC) was first proposed by Akaike for determining the best model among models and avoiding over-fitting (Akaike, 1974). Hurvich and Tsai improved this method and proposed AICc to compensate for the number of data points, and this improved method has been tested valuable in small samples (Hurvich and Tsai, 1989). The equation for calculating the AICc is as listed below:

(4) $$AICc=2*k+N*\ln\left(\frac{RSS}{N}\right)+\frac{2*k*(k+1)}{N-k-1}$$

where *k* is the number of free parameters of models, *N* is the number of points used for fitting, and *RSS* is the RSS from fitting (Hurvich and Tsai, 1989; Jambor et al., 2015).

Besides, we also compared the models with a leave-one-out test, in order to confirm that the models were correctly ranked by the AICc (Bourne et al., 2014). The predicted residual sum of squares (PRESS) is an index of this method first proposed by Allen for model selection (Allen, 1974).

### Image processing and analysis

The DWI images were realigned by using SPM12 (available at: www.fil.ion.ucl.ac.uk/spm). Then, these images were analyzed by using programs written in MatLab (MatLab 2009b; MathWorks, Natick, MA). The conventional bi- and tri-exponential models and the modified tri-exponential model were all used for curve fitting.

Curve fittings of these three models were performed using DWI maps obtained with the first 16 *b*-values (excluding *b* = 8,000 s/mm^2^), and were implemented voxel-by-voxel using the steepest descent algorithm (Lenglet et al., 2009). Signal values were all normalized to corresponding signal value *S*_0_ prior to model fitting. The initial values of parameters were set empirically. For these three models, the initial value of *f*_*slow*_ was set to 0.50, with *ADC*_*slow*_ set to 600 × 10^−6^ mm^2^/s and *ADC*_*fast*_ set to 2,000 × 10^−6^ mm^2^/s. For the modified tri-exponential model, the initial value of *f*_0_ was set to 0.10, while for the conventional tri-exponential model, the initial value of *f*_*very*−*slow*_ was set to 0.10, with *ADC*_*very*−*slow*_ set to 100 × 10^−6^ mm^2^/s. The detail description of the programmed algorithm of model fit is shown in Supplement Figure S1. Thus, the parametric maps and the maps of residual sums of squares (RSS) were derived. Subsequently, the AICc maps of these three models were calculated from the RSS maps. Besides, the PRESS maps were also obtained.

After the curve fitting, the signal intensities at *b* = 8,000 s/mm^2^ were predicted, and the error of the prediction was squared to form the squared prediction error (SPE) by Equation (5). Thus, the SPE maps were derived. The SPE was a new index presented in the current study to evaluate the ability of models in predicting DWI signals at ultra-high *b*-values.

(5) $$SPE={\left(S_{m}-S_{p}\right)}^{2}$$

where *S*_*m*_ and *S*_*p*_ represent the measured signal intensity and the predicted signal intensity at *b* = 8,000 s/mm^2^, respectively.

The regions of interest (ROIs) were drawn by an experienced neuroradiologist. Each ROI was drawn to include corresponding zones as much as possible at one section. Two ROIs were drawn in the cingulate gyrus and supramarginal gyrus, representing gray matter. Six ROIs were drawn in the genu of the corpus callosum, splenium of the corpus callosum, posterior limbs of the internal capsule, centrum semiovale, forceps minor and forceps major, representing white matter. Average values of the parameters and indexes were calculated within each ROI. Besides, the signal-to-noise ratios (SNR) of DWI maps were determined by using a “difference method,” which is based on the evaluation of a difference image of two repeated acquisitions on a single volunteer (Dietrich et al., 2007). ROIs were placed on centrum semiovale and cingulate gyrus, representing for white matter and gray matter, respectively.

In addition, we also performed a stability experiment to investigate the fitting stability of the modified tri-exponential model toward initial conditions. We set random values between 0.05 and 0.10 to initial *f*_0_ values, random values between 0.50 and 0.60 to initial *f*_*slow*_ values, random values between 500 and 800 mm^2^/s to initial *ADC*_*slow*_ values and random values between 1,900 and 2,200 mm^2^/s to initial *ADC*_*fast*_ values. Thus, we totally generated 20 random initial value sets for the modified tri-exponential model. For each initial value set, curve fitting of the modified tri-exponential model was performed, and the mean values of the parameters were calculated over the ROI on the genu of the corpus callosum (CCG) for one volunteer.

### Statistical analysis

All statistical analyses were performed using SPSS version 22 (SPSS Inc, Chicago, IL, USA). The median values and quartile ranges of the RSS, SPE, and AICc were calculated. Wilcoxon signed-rank test were used to compare these three indexes between any pair of the models. A value of *p* < 0.05 was regarded as statistically significant.

## Results

**Figure 2:**
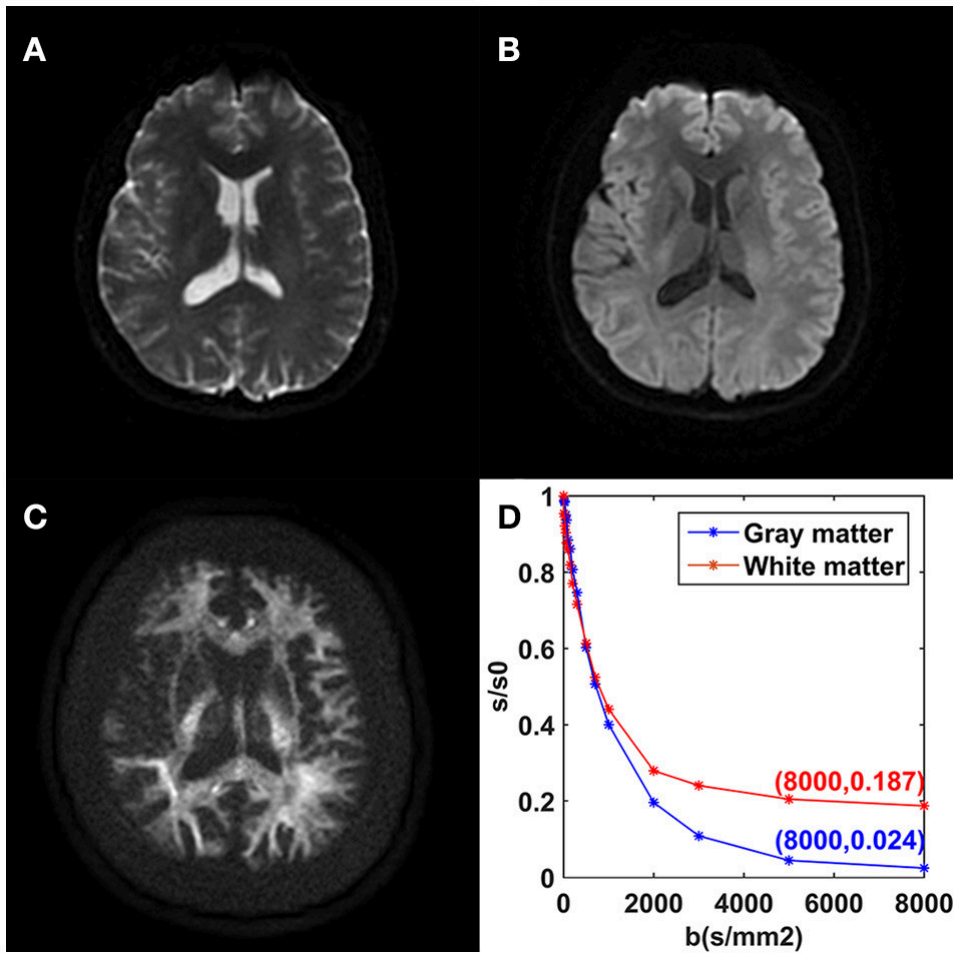
DWI images achieved with *b* = 0 **(A)**, *b* = 1,000 **(B)**, and *b* = 8,000 s/mm^2^ **(C)** from one volunteer. Plots of the average signal-intensity decay of two ROIs as a function of *b* **(D)**. CG, cingulate gyrus; CCg, genu of the corpus callosum.

The mean DWI signal intensity over an ROI decayed much more slowly in white matter than in gray matter, shown in Figure 2. In particular, the remaining signal intensity ratio at *b* = 8,000 s/mm^2^ was as high as 18.7% in the white matter ROI, while only 2.4% in the gray matter ROI. In white matter, the SNRs of the DWI image at *b* = 0, 5,000, and 8,000 s/mm^2^ were 28.2, 27.8, and 23.7, respectively. In gray matter, those were 31.2, 9.8, and 6.5, respectively. For the modified tri-exponential model, the stability experiment showed that the distributions (mean ± *SD*) of the mean *f*_0_, *f*_*slow*_, *f*_*fast*_, *ADC*_*slow*_, and *ADC*_*fast*_ values on CCG over the initial value sets were 18.2 ± 0.6, 58.4 ± 1.2, 23.6 ± 1.2%, 816 ± 7 and 4,525 ± 88 mm^2^/s, respectively.

For the white matter ROIs, the RSSs of the modified tri-exponential model were lower than those of the other two models (*p* < 0.05). Besides, the RSSs of the conventional tri-exponential model were lower than those of the bi-exponential model (*p* < 0.05), shown in Table 1. For the gray matter ROIs, the RSSs of the conventional tri-exponential model were lower than those of the other two models, and there were no significant differences in RSSs between the modified tri-exponential model and the bi-exponential model.

**Table 1:** Residual sums of squares (RSSs) of the bi-exponential, conventional tri-exponential, and modified tri-exponential models in ROIs.

**ROI**	**Bi-exponential (×10^−4^)**	**Conventional tri-exponential (×10^−4^)**	**Modified tri-exponential (×10^−4^)**
CCg	194 (135, 214)	136 (122, 175)^*^	126 (110, 159)^*^^#^
CCs	121 (106, 159)	94 (82, 134)^*^	85 (78, 129)^*^^#^
ICp	61 (45, 72)	47 (34, 59)^*^	44 (29, 51)^*^^#^
CS	64 (58, 86)	50 (44, 70)^*^	44 (38, 66)^*^^#^
Fmi	80 (71, 107)	65 (53, 88)^*^	62 (43, 80)^*^^#^
Fmj	64 (54, 93)	52 (41, 70)^*^	45 (38, 60)^*^^#^
CG	187 (88, 291)	178 (82, 278)^*^	189 (90, 314)^#^
SpG	101 (69, 147)	93 (62, 142)^*^	96 (71, 151)^#^

*CCg, genu of the corpus callosum; CCs, splenium of the corpus callosum; ICp, posterior limbs of the internal capsule; CS, centrum semiovale; Fmi, forceps minor; Fmj, forceps major; CG, cingulate gyrus; SpG, supramarginal gyrus. Data expressed as median (Q1, Q3)*.

* *p < 0.05, vs. the bi-exponential model*;

# *p < 0.05, vs. the conventional tri-exponential model*.

The AICcs of the conventional tri-exponential model were significantly larger than those of the other two models in all ROIs (*p* < 0.05), shown in Table 2. The AICcs of the modified tri-exponential model were lower than those of the bi-exponential model in the white matter ROIs (*p* < 0.05, except for the genu of the corpus callosum), while were higher than those of the bi-exponential model in the gray matter ROIs (*p* < 0.05).

**Table 2:** Small-sample corrected Akaike information criterions (AICcs) of the bi-exponential, conventional tri-exponential and modified tri-exponential models in ROIs.

**ROI**	**Bi-exponential**	**Conventional tri-exponential**	**Modified tri-exponential**
CCg	−102 (−106, −94)	−98 (−103, −89)^*^	−105 (−108, −94)^#^
CCs	−112 (−115, −107)	−108 (−112, −105)^*^	−114 (−117, −110)^*^^#^
ICp	−123 (−126, −118)	−120 (−125, −115)^*^	−126 (−131, −121)^*^^#^
CS	−121 (−124, −118)	−119 (−121, −116)^*^	−124 (−127, −121)^*^^#^
Fmi	−120 (−124, −117)	−117 (−123, −114)^*^	−122 (−128, −119)^*^^#^
Fmj	−123 (−124, −115)	−121 (−122, −113)^*^	−126 (−127, −117)^*^^#^
CG	−112 (−119, −98)	−105 (−113, −91)^*^	−109 (−115, −94)^*^^#^
SpG	−117 (−121, −102)	−110 (−115, −104)^*^	−114 (−118, −107)^*^^#^

*CCg, genu of the corpus callosum; CCs, splenium of the corpus callosum; ICp, posterior limbs of the internal capsule; CS, centrum semiovale; Fmi, forceps minor; Fmj, forceps major; CG, cingulate gyrus; SpG, supramarginal gyrus. Data expressed as median (Q1, Q3)*.

* *p < 0.05, refers to the bi-exponential model*;

# *p < 0.05, refers to the conventional tri-exponential model*.

The PRESSs of the modified tri-exponential model were significantly lower than those of the other two models in all white matter ROIs (*p* < 0.05), while those of the conventional tri-exponential model were significantly lower than those of the other two models in two gray matter ROIs (*p* < 05), shown in Table 3.

**Table 3:** Predicted error sums of squares (PRESS) of the bi-exponential, conventional tri-exponential and modified tri-exponential models in ROIs.

**ROI**	**Bi-exponential (×10^−5^)**	**Conventional tri-exponential (×10^−5^)**	**Modified tri-exponential (×10^−5^)**
CCg	2,930 (2,534, 3,864)	2,399 (1,786, 3,373)^*^	1,985 (1,458, 3,054)^*^^#^
CCs	1,679 (1,593, 2,327)	1,316 (1,121, 1,805)^*^	1,144 (1,044, 1,602)^*^^#^
ICp	861 (685, 1,038)	670 (530, 810)^*^	518 (489, 694)^*^^#^
CS	879 (793, 113)	715 (589, 835)^*^	582 (495, 759)^*^^#^
Fmi	1,006 (736, 1,203)	780 (572, 916)^*^	688 (454, 852)^*^^#^
Fmj	869 (728, 1,243)	619 (546, 886)^*^	573 (434, 807)^*^^#^
CG	1,964 (976, 3,433)	1,898 (915, 3,324)^*^	1,925 (989, 3,510)^#^
SpG	1,146 (819, 1,684)	1,073 (756, 1,621)^*^	1,085 (860, 1,721)

*CCg, genu of the corpus callosum; CCs, splenium of the corpus callosum; ICp, posterior limbs of the internal capsule; CS, centrum semiovale; Fmi, forceps minor; Fmj, forceps major; CG, cingulate gyrus; SpG, supramarginal gyrus. Data expressed as median (Q1, Q3)*.

* *p < 0.05, refers to the bi-exponential model*;

# *p < 0.05, refers to the conventional tri-exponential model*.

**Figure 3:**
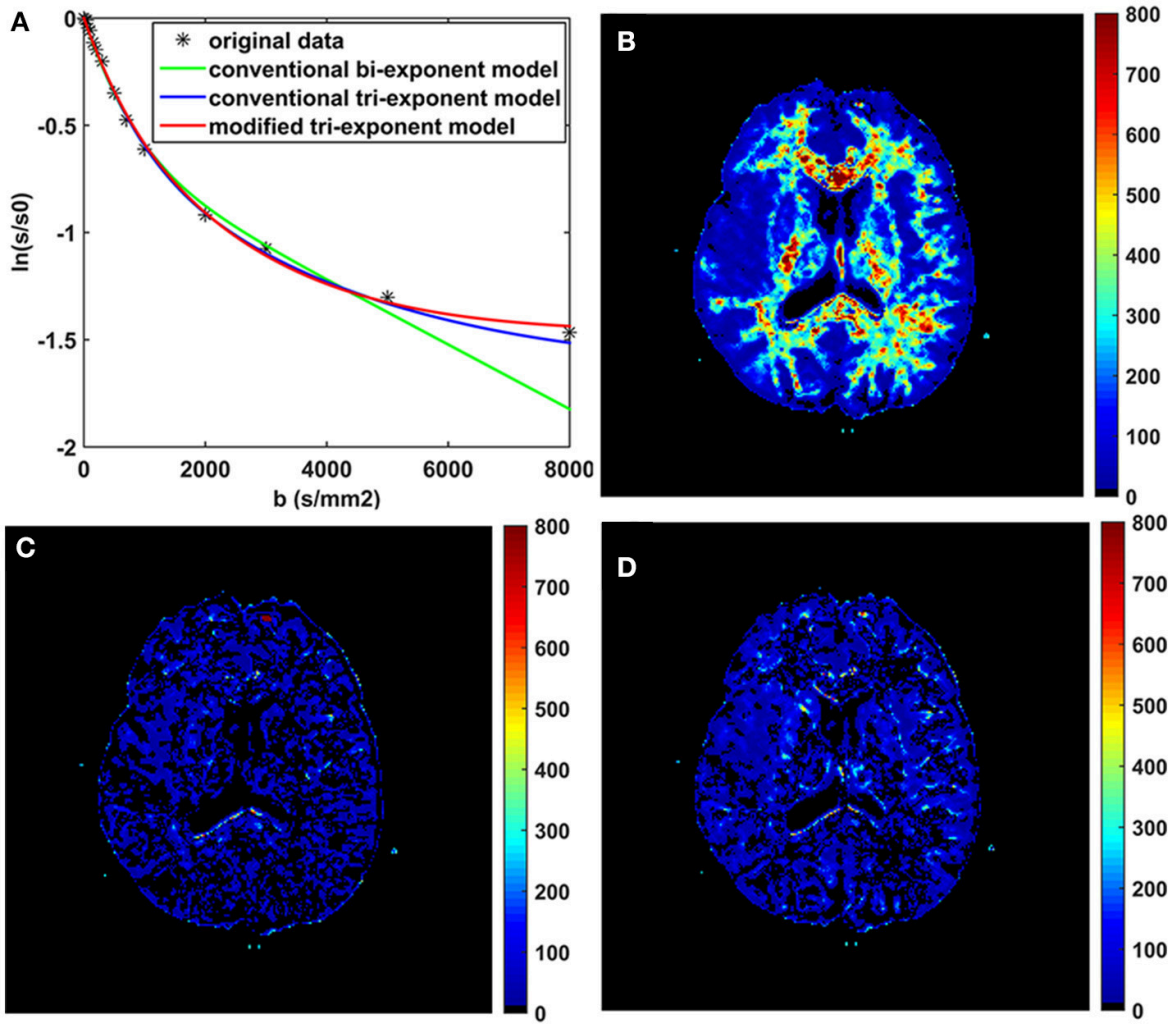
The fitting curves of the three models with the first 16 *b*-values in a typical voxel of white matter **(A)**. The maps of the squared prediction errors (SPE) calculated by the bi-exponential model **(B)**, the conventional tri-exponential model **(C)**, and the modified tri-exponential model **(D)**. The unit for SPE is ×10^−5^.

The bi-exponential model was unable to predict the DWI signal at *b* = 8,000 s/mm^2^ as accurately as the other two models, shown in Figure 3. The SPEs of the bi-exponential model were significantly higher than those of the other two models in all white matter ROIs (*p* < 0.05), shown in Table 4.

**Table 4:** Squared prediction errors (SPEs) of the bi-exponential, conventional tri-exponential and modified tri-exponential models in ROIs of white matter.

**ROI**	**Bi-exponential (×10^−5^)**	**Conventional tri-exponential (×10^−5^)**	**Modified tri-exponential (×10^−5^)**
CCg	500 (340, 595)	56 (31, 69)^*^	46 (22, 60)^*^
CCs	613 (462, 761)	52 (46, 110)^*^	50 (42, 185)^*^
ICp	414 (352, 488)	38 (22, 43)^*^	44 (31, 56)^*^^#^
CS	362 (267, 428)	38 (24, 95)^*^	46 (36, 104)^*^^#^
Fmi	356 (337, 403)	23 (12, 31)^*^	28 (20, 42)^*^^#^
Fmj	328 (293, 351)	24 (17, 31)^*^	33 (23, 40)^*^^#^

*CCg, genu of the corpus callosum; CCs, splenium of the corpus callosum; ICp, posterior limbs of the internal capsule; CS, centrum semiovale; Fmi, forceps minor; Fmj, forceps major. Data expressed as median (Q1, Q3)*.

* *p < 0.05, refers to the bi-exponential model*;

# *p < 0.05, refers to the conventional tri-exponential model*.

**Figure 4:**
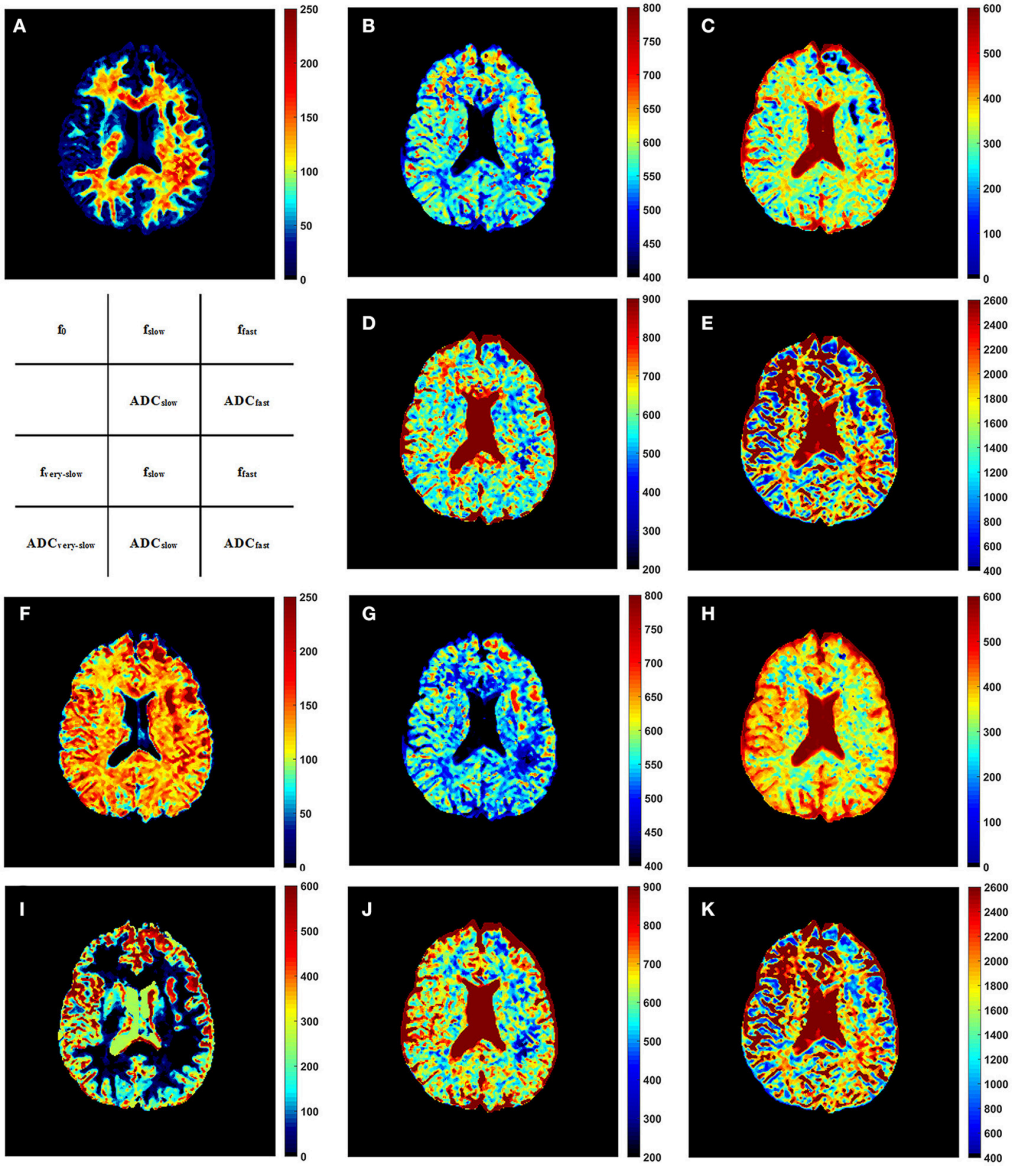
The *f*_0_ map **(A)**, *f*_*slow*_ map **(B)**, *f*_*fast*_ map **(C)**, *ADC*_*slow*_ map **(D)**, and *ADC*_*fast*_ map **(E)** derived from the modified tri-exponential model. The *f*_*very*−*slow*_ map **(F)**, *f*_*slow*_ map **(G)**, *f*_*fast*_ map **(H)**, *ADC*_*very*−*slow*_ map **(I)**, *ADC*_*slow*_ map **(J)**, and *ADC*_*fast*_ map **(K)** derived from the conventional tri-exponential model. The unit for *f* maps is ‰, and the unit for *ADC* maps is ×10^−6^ mm^2^/s.

Representative parameter maps derived from the modified tri-exponential model and the conventional tri-exponential model are shown in Figure 4. In the white matter ROIs, the mean *ADC*_*very*−*slow*_ values (1–7 × 10^−6^ mm^2^/s) were extremely low, and the mean *f*_*very*−*slow*_ values (11.8–18.3%) were similar to the mean *f*_0_ values (11.9–18.7%). However, in the gray matter ROIs, the mean *ADC*_*very*−*slow*_ values (251–445 × 10^−6^ mm^2^/s) were not extremely low and the mean *f*_*very*−*slow*_ values (11.9–15.7%) were much higher than the mean *f*_0_ values (1.2–2.7%), shown in Table 5.

**Table 5:** The *f*_0_ derived from the modified tri-exponential models, and the *f*_*very*−*slow*_ and the *ADC*_*very*−*slow*_ derived from the conventional tri-exponential in ROIs.

**ROI**	***f_0_* (%)**	***f_*very*−*slow*_* (%)**	***ADC_*very*−*slow*_* (×10^−6^ s/mm^2^)**
CCg	16.9 ± 2.8	17.8 ± 0.2	7 ± 11
CCs	18.7 ± 2.0	18.3 ± 1.8	2 ± 4
ICp	15.3 ± 0.7	14.3 ± 0.9	1 ± 1
CS	14.9 ± 1.3	14.3 ± 1.1	1 ± 1
Fmi	13.2 ± 1.8	12.8 ± 1.6	4 ± 3
Fmj	11.9 ± 1.3	11.8 ± 0.8	7 ± 5
CgC	1.2 ± 1.0	15.7 ± 3.5	445 ± 144
SpG	2.7 ± 1.3	11.9 ± 1.5	251 ± 83

*CCg, genu of corpus callosum; CCs, splenium of corpus callosum; ICp, posterior limbs of internal capsule; CS, centrum semiovale; Fmi, forceps minor; Fmj, forceps major; CgC, cingulate cortex; SpG, supramarginal gyrus. Data expressed as mean ± sd*.

**Figure 5:**
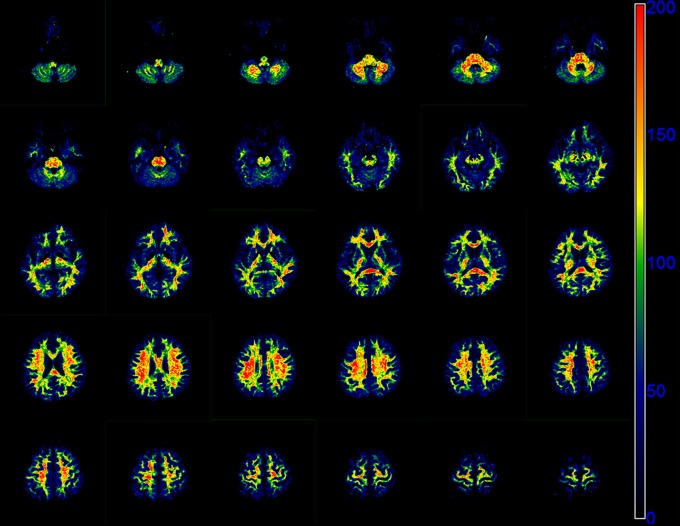
Sequential 30 slices of the *f*_0_ maps of one volunteer derived by the modified tri-exponential model. The *f*_0_ maps are displayed from the top left. The unit is ‰.

Figure 5 presents the whole brain *f*_0_ maps of one volunteer. The *f*_0_ is high in white matter, but very low in gray matter. These images show good resolution and good definition at white-gray matter interfaces.

## Discussion

In our study, the AICcs and PRESSs of the new model were found to be the lowest in white matter, suggesting that this new model fit better than the conventional bi-exponential and tri-exponential models and may provide more detailed information. The *f*_0_ values were found to be very small in gray matter but ranging 10–20% in white matter. This result indicates that the strictly diffusion-limited compartment may be an important component in white matter and may need to be considered when we develop models for multi-*b*-value DWI.

First of all, we certified that the fraction of the strictly diffusion-limited compartment (*f*_0_) in white matter cannot be explained only by noise. In white matter, the remaining signal intensity ratio was as high as 18.7% at *b* = 8,000 s/mm^2^, while the SNR was 23.7. Thus, the ratio of noise at *b* = 8,000 s/mm^2^ to signal at *b* = 0 s/mm^2^ was only 0.79%. This ratio is much lower than the fractions of the strictly diffusion-compartment in white matter which were ranging from 11.8 to 18.7%. Thus, the existence of the strictly diffusion-limited compartment in white matter is not only a result of noise.

In the present study, the bi-exponential model was found to be an over-simplified model. According to the intravoxel incoherent motion (IVIM) theory, the fast ADC is thought to be linked to the microcirculatory perfusion of blood within the capillaries, while the slow ADC is related to diffusion of water molecules in the tissues (Le Bihan and Turner, 1992; Koh et al., 2011; Cercueil et al., 2015). This theory is not suitable when *b* > 1,000 s/mm^2^. Another explanation for this model is that two components represent intra- and extra-cellular compartments, respectively (Niendorf et al., 1996; Steier et al., 2012). However, researchers have found that the attenuation of DWI signal does not obey the mono-exponential model even without extra-cellular compartment or even in a single cell (Grant et al., 2001; Schwarcz et al., 2004; Steier et al., 2012). These findings of previous studies also indicate that the bi-exponential model may not be a satisfying model for explaining the attenuation of DWI signal. Models with more pools might be preferable to accurately reflect the diffusion motion of water molecules in tissues.

In the present study, we also found that the SPEs of the bi-exponential model were much higher than the other two models in white matter. As we know, the remaining signal intensity ratio will be very low at ultra-high *b*-value for compartments with a normal ADC, while this ratio will still remain high for the compartment with an extremely low ADC. Hence, as the bi-exponential model does not contain the compartment with extremely low ADC, it is conceivable that the predicted signal at *b* = 8,000 s/mm^2^ would be much lower than the measured value, resulting in high SPE.

When compared with the modified tri-exponential model, the conventional tri-exponential model had significantly higher AICcs in all ROIs and was considered as an over-fitting model. More importantly, the biological implications of the *ADC*_*very*−*slow*_ compartment differed between white matter and gray matter. In the white matter ROIs, *ADC*_*very*−*slow*_ values were extremely small, and the *f*_*very*−*slow*_ values were found to be similar to the *f*_0_ values. Thus, *f*_0_ and *f*_*very*−*slow*_ represent the fraction of the same compartment with extremely small ADC in white matter. However, when *f*_0_ values were negligible in the gray matter ROIs, the *f*_*very*−*slow*_ values were still as high as in white matter, while the *ADC*_*very*−*slow*_ values were not extremely small. This finding suggests that the *ADC*_*very*−*slow*_ compartment no longer represents the compartment with extremely small ADC in gray matter. In our view, the three compartments of the conventional tri-exponential model may represent three major proton pools in tissues, while the major proton pools may differ among tissues. Hence, the parameters derived from this model may have no specific biological implications. This might be an important limitation for the application of the conventional tri-exponential model. Generally, models with more pools may also suffer from this fatal limitation.

On the contrary, by directly setting the *ADC*_*very*−*slow*_ to zero, the modified tri-exponential model is able to detect the volume fraction of the extremely-low ADC compartment. In the present study, the mean *f*_0_ values were found to be non-ignorable in white matter, ranging from 11.9 to 18.7%. Ferizi et al. also found that three compartment models with a “dot” compartment (zero radius sphere) can produce better fit for diffusion MRI in white matter, suggesting the existence of the extremely-low ADC compartment (Ferizi et al., 2014). In white matter, it has been suspected that there are stationary water molecules trapped in glial cells and other small compartments or bound to membranes and other subcellular structures (Alexander et al., 2010). However, Dhita et al. recently showed that still water compartment was absent in white matte by using isotropic diffusion measurement (Dhital et al., 2017). A similar conclusion was also made by Veraart el al. using single-direction measurements (Veraart et al., 2016). Although Dhital et al. found that the slowly diffusing water pools existed in all directions, these pools were suspected to reside in separate micro-environments (Dhital et al., 2017). It is recommended that orientation dispersion of axons and glial processes should be taken into account when developing models for fitting isotropic diffusion measurement (Dhital et al., 2017). Thus, the exact biological interpretation for the strictly diffusion-limited compartment in white matter needs to be investigated further. Besides, the existence of this compartment in other normal or pathological tissue also needs to be investigated.

One limitation of the application of ultra-high *b*-value DWI is low SNR. To ensure high SNRs, the NSAs were designed very large in this study, especially for DWI images with ultra-high *b*-values. Our result showed that the SNRs of DWI maps with ultra-high *b*-values were comparable with that of DWI maps with *b* = 0 s/mm^2^. However, traditional magnitude reconstruction which is used in this study, may lead to an accumulation of noise (Eichner et al., 2015). Averaging the repeat measurements in complex domain is recommend to further improve the SNR, while it requires complex phase navigation and is not normally provided by hardware vendors (Jones et al., 2013; Eichner et al., 2015).

Anisotropic resolution with a high in-plant resolution and a large slice thickness was applied in this study. Clinically, a high in-plant resolution is required to distinguish fine structure in brain. SNR has a linear relationship with voxel volume, thus a relative large slice thickness can improve the SNR. Besides, a large slice thickness can also shorten the scan time. However, anisotropic resolution can lead to differential averaging of fiber orientations (Jones et al., 2013). This effect is not accounted for in this study because the DWI images used for model fitting do not contain direction information.

There are still several limitations in this study. First, the total scan time of this sequence is too long for clinical practice. Hence, the multi-*b*-value DWI sequence should be optimized, including the selection of *b*-values and NSAs. Second, in gray matter, the SNRs of DWI maps at ultra-high *b*-values were low, which reduced the reliability of some results. However, the main focus of the study was on white matter, and the SNR still remained high at ultra-high *b*-values in white matter. Third, the impact of T2 values of different compartments were not evaluated in our study, and need further research.

In conclusion, the bi-exponential model is an over-simplified model and unable to predict the signal intensity at ultra-high *b*-values in white matter, while the conventional tri-exponential model is an over-fitting model and has no specific biological implication for each compartment. The new model fits better than the other two models, and may provide additional information.

## Author contributions

QZ: Conceived the idea; BJ: Supervised the work; CL and FD: Collected the data; JZ, FD, and FS: Analyzed the data; QZ, FS, and CL: Wrote the main manuscript text. All authors reviewed the manuscript.

### Conflict of interest statement

The authors declare that the research was conducted in the absence of any commercial or financial relationships that could be construed as a potential conflict of interest.
