# Supplementary material for: A Modified Tri-Exponential Model for Multi-b-value Diffusion-Weighted Imaging: A Method to Detect the Strictly Diffusion-Limited Compartment in Brain
Source: Front Neurosci. 2018 Feb 26;12:102. doi: 10.3389/fnins.2018.00102 (PMC5834430; doi:10.3389/fnins.2018.00102)
Supplement: Supplementary file 1 [file DataSheet1.PDF]

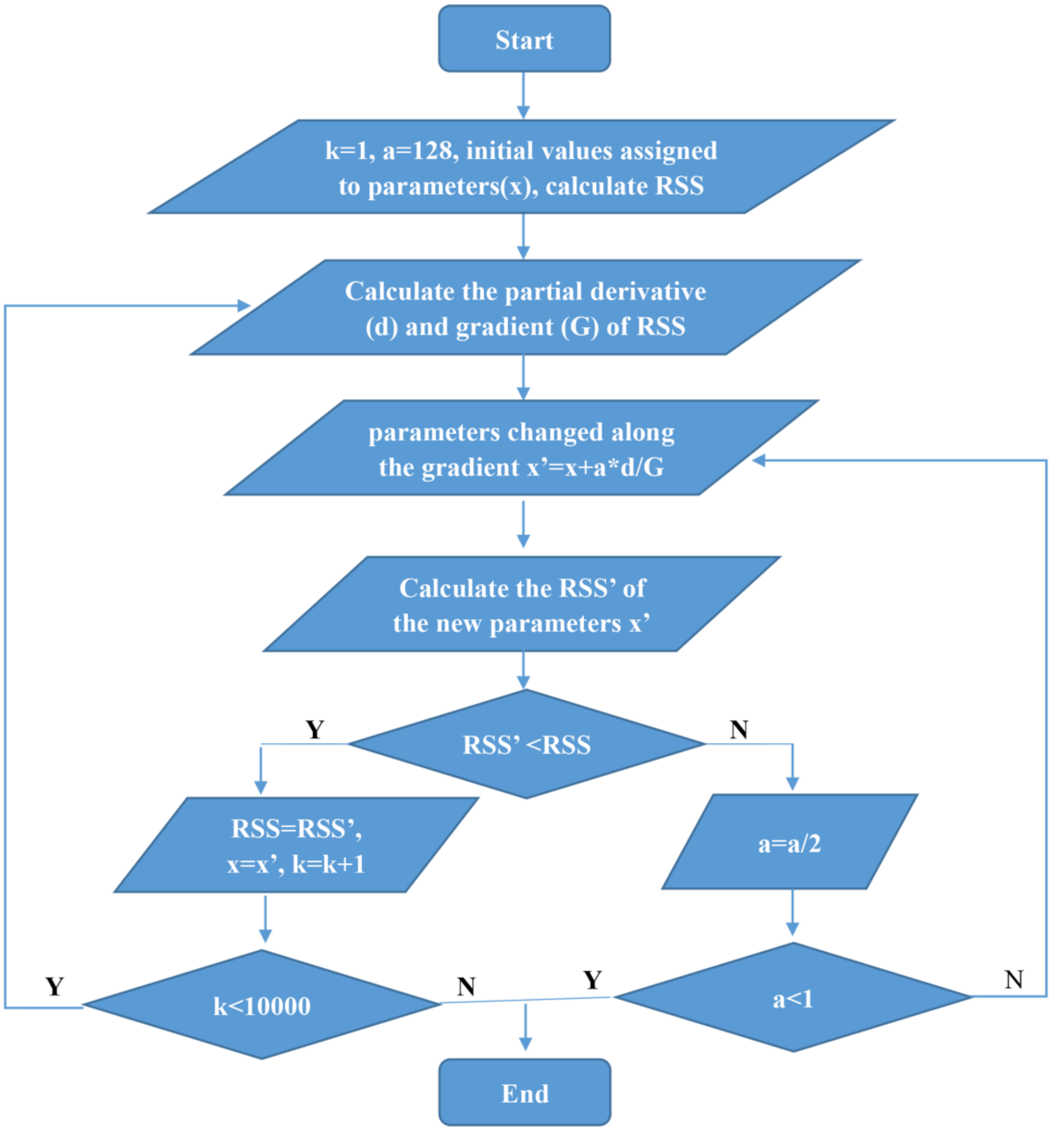

Supplement figure S1. Programmed algorithm of model fit using the steepest descent algorithm. k is a loop variant to avoid too many loops. a is the step size.
